# Therapeutic and research frontiers in fibromyalgia: integrating pathophysiology with innovative drug repurposing

**DOI:** 10.1007/s10787-026-02349-5

**Published:** 2026-07-23

**Authors:** Sheer A. Joodi, Dalia A. Nawwar, Nora O. Abdel Rasheed, Weam W. Ibrahim, Helmy M. Sayed

**Affiliations:** 1 Postgraduate Program in Pharmacology and Toxicology, Faculty of Pharmacy, Cairo University, ElKasr Elaini Street, Cairo, 11562 Egypt; 2 Department of Pharmacology and Toxicology, Faculty of Pharmacy, Cairo University, Cairo, Egypt

**Keywords:** Fibromyalgia, Central sensitization, NLRP3 inflammasome, Microglial polarization, Astrocytic polarization, Mitochondrial dysfunction

## Abstract

Fibromyalgia (FM) is a complex chronic pain syndrome characterized by widespread musculoskeletal pain, fatigue, sleep disturbance, psychological symptoms, and cognitive dysfunction, profoundly impairing quality of life. Despite its multifactorial nature, only a few pharmacological therapies have been approved by the Food and Drug Administration (FDA), and these mainly provide symptomatic relief. Many patients experience inadequate efficacy or intolerable adverse effects, emphasizing the need for further research and improved therapeutic strategies. This review highlights contributing factors in the pathophysiology of FM, including neurochemical alterations, central sensitization, neuroinflammation, oxidative stress, mitochondrial dysfunction, gut microbiota disturbances, and autoimmunity. While some of these factors are well-established, others remain under investigation. Therapeutic strategies are discussed alongside repurposed drugs in preclinical and clinical studies, including N-methyl-D-aspartate (NMDA) receptor antagonists, neurokinin-1 receptor antagonists, drugs targeting the gamma-aminobutyric acid (GABA) system, antiepileptics, antidepressants, opioids, cannabinoids, dopamine receptor agonists, melatonin receptor agonists, and antidiabetics. Future research frontiers in FM should focus on addressing comorbidities and targeting central sensitization by enhancing descending inhibitory pain pathways, suppressing neuroinflammation through NOD-like receptor protein 3 (NLRP3) inflammasome inhibition and promotion of anti-inflammatory glial polarization besides attenuating oxidative stress and mitochondrial dysfunction. Moreover, repurposing drugs from related pain conditions such as migraine and neuropathic pain offers new therapeutic opportunities. Accordingly, this multi-target strategy may facilitate the development of effective therapies for FM.

**Graphical abstract:**

**Figure:**
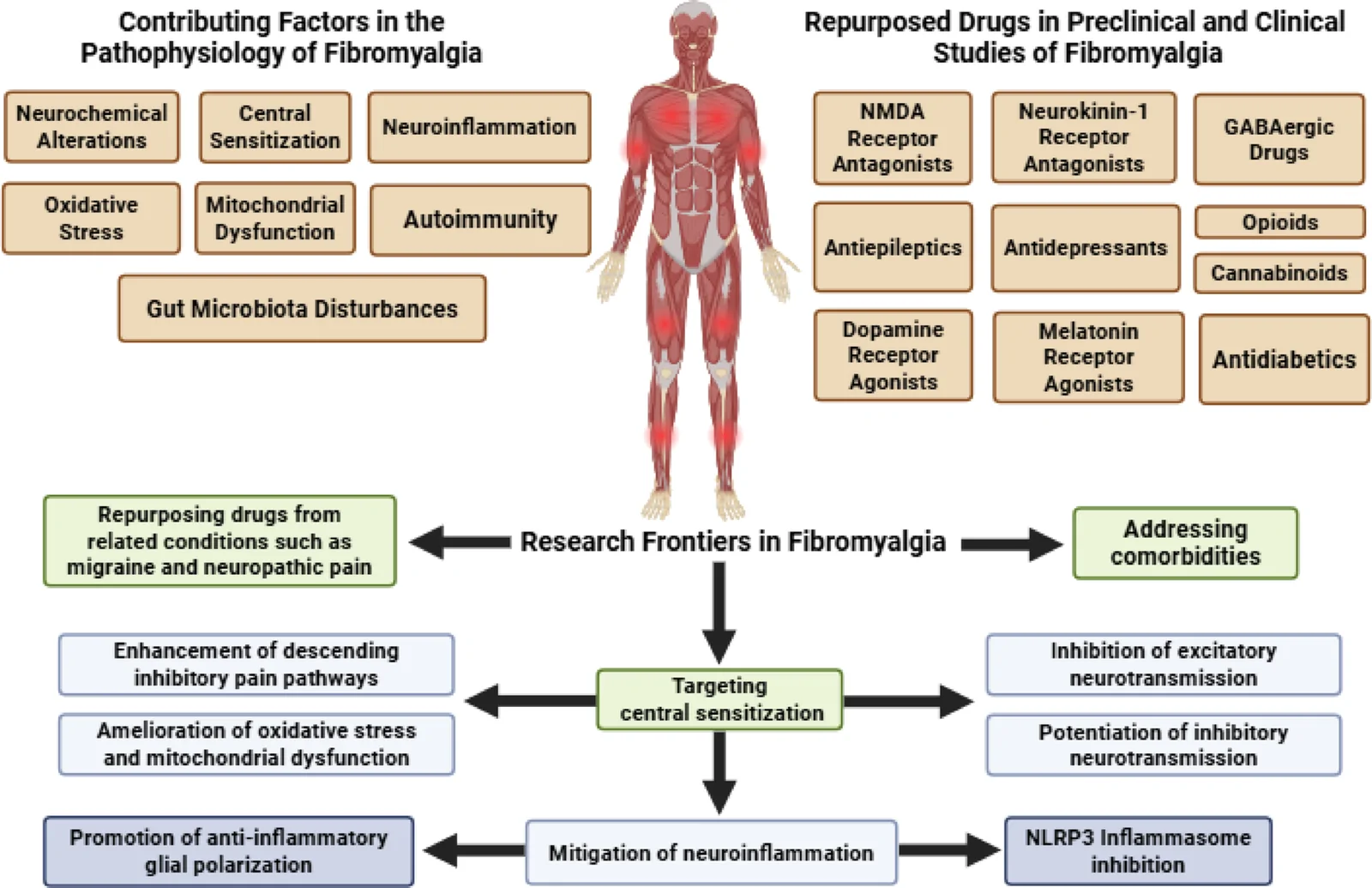

## Introduction

Nociplastic pain is a chronic condition associated with central sensitization, in which pain signaling is amplified, resulting in hyperalgesia reflected as an exaggerated response to stimuli that normally cause pain and allodynia manifested as pain triggered by stimuli that are not usually painful despite the absence of obvious tissue damage or underlying disease. Examples of nociplastic pain include fibromyalgia (FM), migraine, and irritable bowel syndrome (Cavalli et al. 2019; Atta et al. 2023a, b). FM is a chronic musculoskeletal condition marked by pain, stiffness, and tenderness in the muscles, tendons, and joints. It is characterized by widespread pain, often experienced in the arms, legs, chest, abdomen, and back, leading to significant impairment in daily activities (Siracusa et al. 2021; Gyorfi et al. 2022; Hsiao et al. 2025). The earliest description of FM dates back to the 19th century. The term “fibrositis,” introduced by Gowers in 1904, remained widely used until the 1970s and 1980s, when hypotheses began to shift toward a central nervous system (CNS)-related etiology. In 1950, Graham described fibrositis as a pain syndrome lacking a clearly identifiable organic pathology. Later, in the mid-1970s, Smythe and Moldofsky introduced the term “FM” and identified tender points, which are localized areas characterized by hyperalgesia and allodynia (Siracusa et al. 2021; Gyorfi et al. 2022). FM affects 2–4% of the adult population, predominantly occurring in middle-aged women with a female-to-male ratio of 3:1, although it can affect individuals of any age in both sexes (Abdelrheem et al. 2026; Osama et al. 2026).

In 1990, the American College of Rheumatology (ACR) defined the initial diagnostic criteria for FM, which were subsequently modified in 2010 and 2011. According to the 2016 revised ACR criteria, diagnosis no longer relies on tender points evaluation, shifting instead to a combination of widespread pain and symptom severity. To establish a diagnosis, four essential criteria are required. First, the presence of generalized pain, defined as pain in at least 4 of 5 regions. Second, symptoms persist at a similar level for at least three months. Third, the Widespread Pain Index (WPI) and Symptom Severity Scale (SSS) are used to assess the number of painful body regions and comorbidities, respectively, with diagnostic thresholds of WPI ≥ 7 and SSS ≥ 5, or alternatively WPI of 4–6 combined with SSS ≥ 9. Finally, the diagnosis of FM remains valid irrespective of coexisting conditions and does not exclude the presence of other clinically important illnesses (Wolfe et al. 2010, 2016).

Notably, FM is frequently associated with comorbidities, such as sleep disturbance, chronic fatigue, anxiety, depression, and cognitive impairment (fibro fog), and it has also been linked to various conditions, including diabetes, rheumatic diseases, psychiatric and neurological disorders, as well as hypothalamic-pituitary-adrenal axis dysfunction (Siracusa et al. 2021; Atta et al. 2023a, b). Moreover, various infections have been implicated in the onset of FM, comprising Herpesvirus, Epstein–Barr virus, Lyme disease, and COVID-19 (Pridgen et al. 2017; Findeisen et al. 2025). In FM, the dorsal root ganglia (DRG) and dorsal horn of the spinal cord are crucial regions involved in pain processing; however, the dorsal horn represents the key site for the initiation of central sensitization. Excitatory neurotransmitters like glutamate enhance synaptic transmission in these regions and amplify pain signaling through the ascending pathway, transmitting pain signals from the spinal cord to the brain (Atta et al. 2023a, b; Osama et al. 2026). The thalamus acts as a relay station in the spinothalamic pathway and contributes to the amplification and transmission of pain signals to cortical regions (Atta et al. 2023a, b; Mohamed et al. 2025). Although the exact pathophysiological mechanism of FM remains unclear, several contributing factors have been involved, including genetic predisposition, neurochemical alterations, central sensitization, neuroinflammation, oxidative stress, mitochondrial dysfunction, gut microbiota disturbances, and autoimmunity (Siracusa et al. 2021; Atta et al. 2023a, b; Findeisen et al. 2025; Ho et al. 2025).

This article will address contributing factors in the pathophysiology of FM, with a particular focus on central sensitization and neuroinflammation, discuss therapeutic strategies, and highlight repurposed drugs in preclinical and clinical studies, thereby linking pharmacological mechanisms to disease-relevant pathophysiological pathways.

## Methodology

This narrative review was conducted using PubMed and Scopus databases, in addition to Google Scholar and ClinicalTrials.gov. The search used the following keywords: FM, central sensitization, neuroinflammation, oxidative stress, mitochondrial dysfunction, gut dysbiosis, and drug repurposing. Original research articles, narrative reviews, and systematic reviews published up to 2026 were selected based on relevance to contributing factors in the pathophysiology, therapeutic strategies, and repurposed drugs in preclinical and clinical studies of FM.

## Contributing factors in the pathophysiology of fibromyalgia

### Neurochemical alterations

In the descending pain pathways, the brain modulates pain perception by releasing inhibitory neurotransmitters, including serotonin, norepinephrine, and endorphins. These neurotransmitters run down to the spinal cord, where they suppress the transmission of pain signals (Atta et al. 2023a, b). The primary changes observed in FM involve disruptions in monoaminergic neurotransmission, characterized by reduced levels of serotonin and norepinephrine, resulting in impaired descending inhibitory pain pathways and elevated levels of glutamate and substance P (SP) in the spinal cord. Further, dopamine dysregulation and altered activity of endogenous cerebral opioids have also been observed. Collectively, these alterations contribute to central sensitization (Siracusa et al. 2021; Atta et al. 2023a, b).

### Central sensitization

Central sensitization primarily occurs in the spinal dorsal horn and brainstem, may persist for months to years, and contributes to both neuropathic and nociplastic pain (Atta et al. 2023a, b; Ma et al. 2024). In response to sensory input, glutamate is released from sensory afferents and activates α-amino-3-hydroxy-5-methyl-4-isoxazolepropionic acid (AMPA) receptor, initiating depolarization of spinal dorsal horn neurons. Meanwhile, activation of NMDA receptor by glutamate promotes synaptic plasticity through Ca²⁺-dependent signaling pathways, thus driving central sensitization (D’Mello and Dickenson 2008; Atta et al. 2023a, b). Additionally, SP is co-released with glutamate from C fibers and binds to neurokinin-1 receptor (NK-1R) on postsynaptic dorsal horn neurons, facilitating NMDA receptor activity and enhancing the transmission of pain signals to the brain (D’Mello and Dickenson 2008; Siracusa et al. 2021). Reduced gamma-aminobutyric acid (GABA)-mediated inhibitory tone in the spinal dorsal horn further contributes to the persistence of this sensitized state (Atta et al. 2023a, b). Moreover, activated glial cells release brain-derived neurotrophic factor (BDNF), which binds to tropomyosin receptor kinase B on secondary neurons and activates downstream intracellular signaling pathways, thereby promoting synaptic plasticity and central sensitization (Atta et al. 2023a, b; Ma et al. 2024).

### Neuroinflammation

**Fig. 1:**
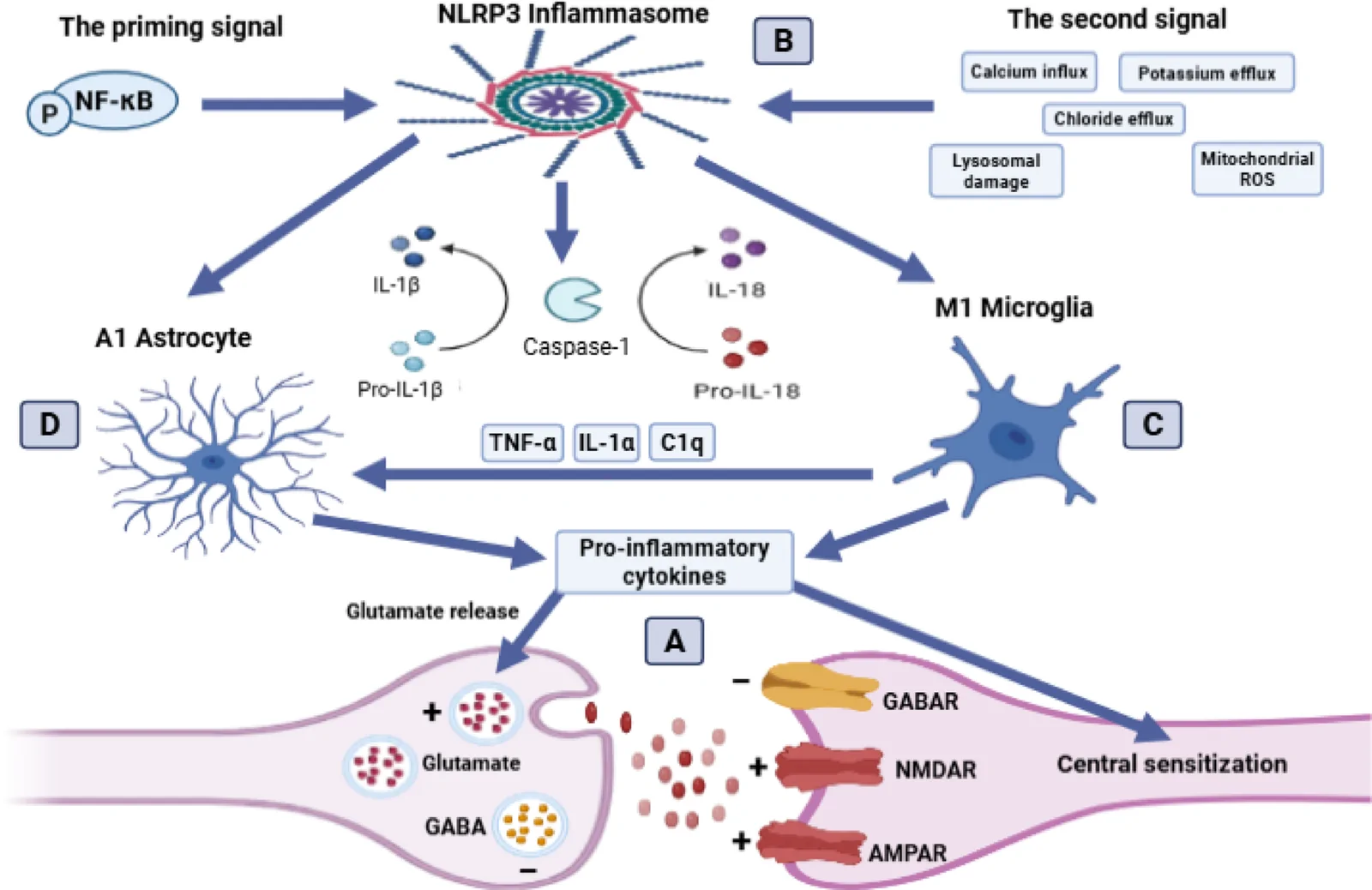
**A** Role of pro-inflammatory cytokines in central sensitization, **B** NLRP3 inflammasome activation, **C** Role of M1 microglia in central sensitization and A1 astrocytic activation, and **D** Role of A1 astrocyte in sustaining central sensitization in association with M1 microglia. NLRP3 NOD-like receptor protein 3, NF-κB Nuclear factor-kappa B, TNF-α Tumor necrosis factor-alpha, IL-1β Interleukin-1β, IL-18 Interleukin-18, AMPAR α-amino-3-hydroxy-5-methyl-4-isoxazolepropionic acid receptor, NMDAR N-methyl-D-aspartate receptor, GABAR Gamma-aminobutyric acid receptor

Pro-inflammatory cytokines, such as tumor necrosis factor-alpha (TNF-α), interleukin IL-1β (IL-1β), and IL-18 contribute to central sensitization by various mechanisms, including promotion of glutamate release, potentiation of AMPA and NMDA receptors excitatory signaling, and suppression of GABAergic inhibitory transmission in the spinal dorsal horn (Ji et al. 2019; Donnelly et al. 2020; Atta et al. 2023a, b) **(**Fig. 1A**)**. Elevated plasma levels of TNF-α and IL-6 have been reported in patients with FM (Ernberg et al. 2018). In this regard, Toll-like receptor-4 (TLR4) activation enhances nuclear factor-kappa B (NF-κB) activity, which upregulates the transcription of NOD-like receptor protein 3 (NLRP3) and pro-inflammatory cytokines (Lai et al. 2023; Zhan et al. 2023; Joodi et al. 2025). Further, downregulation of TLR4 by eicosapentaenoic acid and electroacupuncture has been associated with reduced pain hypersensitivity in a mouse model of intermittent cold stress-induced FM (Lai et al. 2023; Hsiao et al. 2025).

#### NLRP3 inflammasome

It is among the most widely studied inflammasomes, comprising the NLRP3 sensor, the apoptosis-associated speck-like protein containing CARD (ASC) adaptor, and the caspase-1 protease (Zhao et al. 2024). NLRP3 is primarily expressed by immune cells, including microglia, macrophages, dendritic cells, and neutrophils (D’Amico et al. 2021). The canonical activation of NLRP3 inflammasome requires two distinct signals. The first signal involves a priming step, during which NF-κB upregulates the gene expression of NLRP3, pro-IL-1β, and pro-IL-18. Next, the second signal is triggered by various upstream events, such as calcium influx, potassium efflux, chloride efflux, mitochondrial reactive oxygen species (ROS) production, and lysosomal damage, resulting in inflammasome assembly and subsequent activation of caspase-1, which cleaves pro-IL-1β and pro-IL-18 into their active forms (D’Amico et al. 2021; Zhan et al. 2023) (Fig. 1B). Experimentally, the administration of BMS-986,299, an NLRP3 agonist, induced pain hypersensitivity, depression-like behaviors, sleep disturbance, and glial cells activation in the spinal cord of reserpine-treated mice, a model of FM. Conversely, NLRP3 knockout reversed these effects (Zhao et al. 2024). Additionally, downregulation of NLRP3 by astaxanthin, myricitrin, suramin, and brilliant blue G has been associated with improvement in FM-like symptoms in a rodent model of reserpine-induced FM (D’Amico et al. 2021; Aboutaleb et al. 2024; Zhao et al. 2024; Mohamed et al. 2025). Further, activation of NLRP3 inflammasome was observed in blood mononuclear cells (BMCs) from FM patients, accompanied by increased serum levels of IL-1β and IL-18. Moreover, serum IL-1β levels showed a positive correlation with pain intensity in FM (Cordero et al. 2014).

#### Microglial polarization

Microglia are the major phagocytes of the CNS. Activated microglia can be polarized into two phenotypes: classically activated microglia (M1, pro-inflammatory) and alternatively activated microglia (M2, anti-inflammatory). At the level of spinal cord, several agonist-receptor interactions are implicated in the activation of M1 microglia, including adenosine triphosphate (ATP)-purinergic receptors, SP-NK-1R, lipopolysaccharide-TLR4, and chemokines-chemokine receptors (Zhu et al. 2021; Atta et al. 2023a, b). These interactions promote the activation of microglial mitogen-activated protein kinases (MAPKs), especially p38 MAPK, thus contributing to central sensitization through the production of pro-inflammatory cytokines, chemokines, and BDNF (Ji and Suter 2007; Yang et al. 2022; Atta et al. 2023a, b) **(**Fig. 1C**)**. In contrast, M2 microglia produce anti-inflammatory cytokines, like IL-4 and IL-10 (Atta et al. 2023a, b). Accordingly, microglial polarization toward the M2 phenotype induced by galantamine and suramin has been associated with amelioration of FM-like symptoms in a rat model of reserpine-induced FM (Atta et al. 2023a, b; Mohamed et al. 2025). Further, an imbalance in the typical M1/M2 pattern has been reported in patients with nociplastic pain disorders, including FM (Tripathi et al. 2020; Atta et al. 2023a, b).

#### Astrocytic polarization

Astrocytes, star-shaped glial cells in the CNS, play a central role in FM-like pain behaviors. Reactive astrocytes can be polarized into two phenotypes: the classically activated A1 phenotype, which is associated with pro-inflammatory and neurotoxic effects, and the alternatively activated A2 phenotype, which enhances neuroprotection (Al-Matarneh et al. 2025). M1 microglia promote the initiation of FM-like pain behaviors in the spinal dorsal horn, while A1 astrocytes facilitate the persistence of chronic pain together with M1 microglia. Mechanistically, M1 microglia release TNF-α, IL-1α, and complement component C1q, driving astrocytic polarization toward the A1 phenotype (Lu and Gao 2023; Al-Matarneh et al. 2025). These pro-inflammatory stimuli activate extracellular signal-regulated kinase (ERK) and c-Jun-N-terminal kinase (JNK) signaling pathways in spinal astrocytes, thereby enhancing the production and release of chemokines (Ji et al. 2019). Additionally, A1 astrocytes release pro-inflammatory cytokines that, alongside chemokines, contribute to central sensitization and maintenance of chronic pain (Ji et al. 2019; Donnelly et al. 2020; Al-Matarneh et al. 2025) **(**Fig. 1D**)**. Conversely, A2 astrocytes produce anti-inflammatory cytokines and support neuroprotection. Interestingly, astrocytic polarization toward the A2 phenotype induced by apigenin has been associated with amelioration of hyperalgesia and allodynia in a rat model of reserpine-induced FM (Al-Matarneh et al. 2025).

### Oxidative stress

**Fig. 2:**
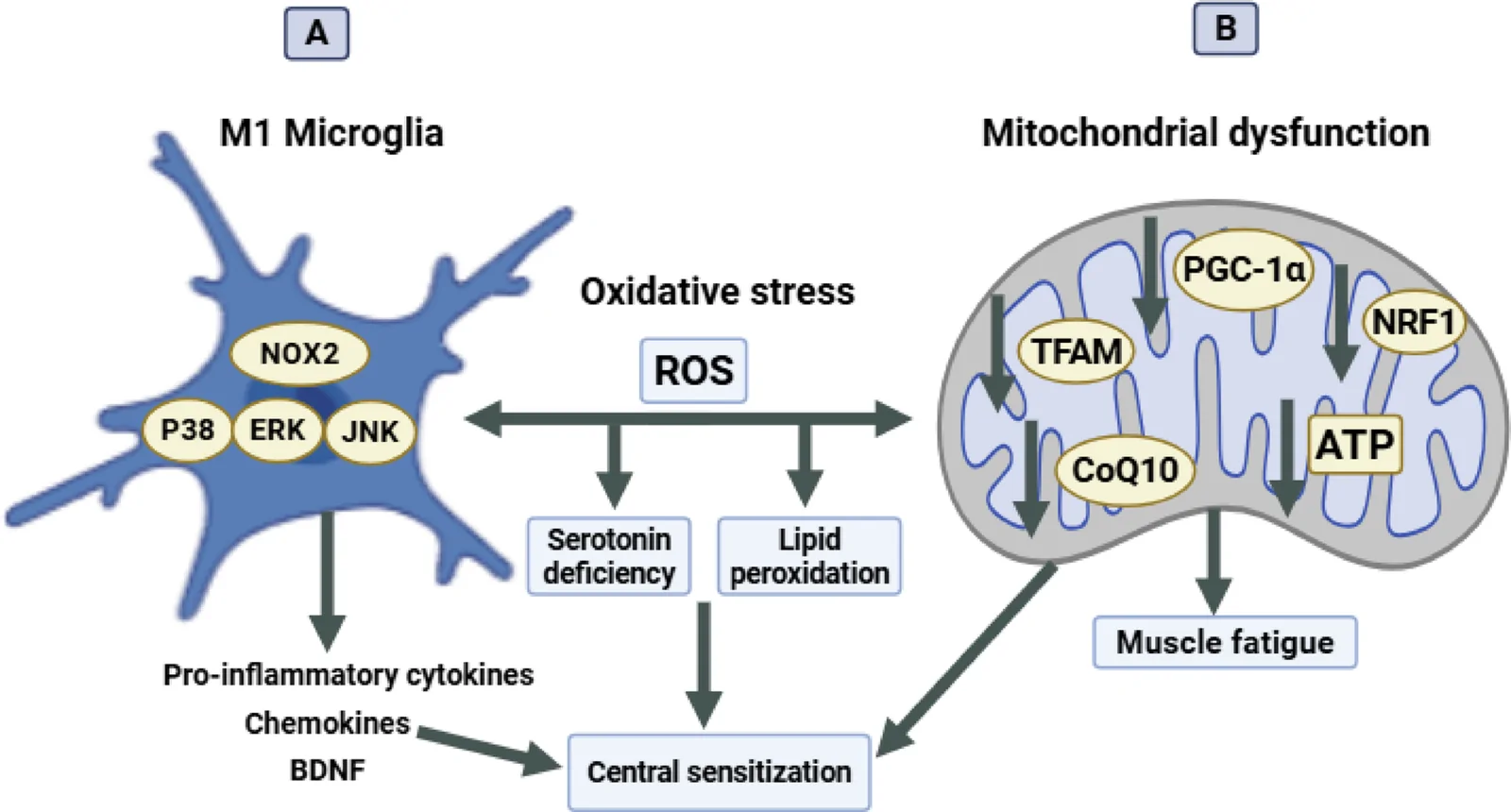
Role of oxidative stress in **A** M1 microglial activation and **B** mitochondrial dysfunction eventually contributing to central sensitization. ATP Adenosine triphosphate, BDNF Brain-derived neurotrophic factor, CoQ10 Coenzyme Q10, ERK Extracellular signal-regulated kinase, JNK c-Jun-N-terminal kinase, NOX2 NADPH oxidase 2, NRF1 Nuclear respiratory factor 1, PGC-1α Peroxisome proliferator-activated receptor-gamma coactivator 1-alpha, ROS Reactive oxygen species, TFAM Mitochondrial transcription factor A

Oxidative stress results from an imbalance between ROS production and antioxidant defense mechanisms, leading to lipid peroxidation, mitochondrial dysfunction, neuroinflammation, and neurotransmitter dysregulation, all of which contribute to pain amplification in FM (Ho et al. 2025). The role of activated microglia in chronic pain states extends beyond the production of pro-inflammatory cytokines to facilitate ROS formation through the upregulation of NADPH oxidase 2 (NOX2) (Paroli et al. 2024; Ho et al. 2025). The resulting oxidative stress may impair serotonin signaling by shunting tryptophan toward the kynurenine pathway, damaging key synthetic enzymes such as tryptophan hydroxylase, and disrupting ATP-dependent neurotransmitter release. These processes promote serotonin depletion, which is implicated in pain hypersensitivity and mood disturbances (Ho et al. 2025) (Fig. 2A). Consistently, malondialdehyde, a marker of lipid peroxidation, and nitric oxide levels were elevated in FM patients and showed a positive correlation with disease severity. In contrast, levels of antioxidant enzymes, including catalase, superoxide dismutase, glutathione reductase, and glutathione peroxidase, were reduced. Notably, only catalase, glutathione reductase, and glutathione peroxidase showed a negative correlation with disease severity (Shukla et al. 2020).

### Mitochondrial dysfunction

Oxidative stress is associated with mitochondrial dysfunction, characterized by impaired electron transport chain activity, excessive ROS production, and decreased ATP synthesis, contributing to muscle fatigue, M1 microglial activation, and central sensitization (Ho et al. 2025). BMCs from FM patients exhibited significant downregulation of key genes involved in mitochondrial biogenesis, including peroxisome proliferator-activated receptor-gamma coactivator 1-alpha (PGC-1α), nuclear respiratory factor 1 (NRF1), and mitochondrial transcription factor A (TFAM), accompanied by reduced coenzyme Q10 (CoQ10) levels, an essential electron carrier in the mitochondrial respiratory chain (Cordero et al. 2012, 2014) (Fig. 2B). In this regard, adenosine monophosphate-activated protein kinase (AMPK) activation induces phosphorylation of PGC-1α, which promotes mitochondrial biogenesis and upregulation of antioxidant enzyme expression. A reduction in phosphorylated AMPK and PGC-1α levels has been demonstrated in fibroblasts from FM patients (Alcocer-Gómez et al. 2015). CoQ10 supplementation ameliorated FM-like symptoms, oxidative stress, and mitochondrial dysfunction in a rat model of reserpine-induced FM (Belviranlı et al. 2026a, b; Belviranlı et al. 2026a, b). Additionally, in a randomized controlled trial (RCT) involving FM patients, CoQ10 supplementation was shown to significantly reduce pain as well as fatigue and to restore antioxidant enzymes and mitochondrial biogenesis *via* AMPK phosphorylation (Cordero et al. 2013). Further, CoQ10 supplementation in pregabalin-treated FM patients provided additional benefits compared with pregabalin alone, resulting in greater reductions in pain, anxiety, and brain activity, as well as improvements in mitochondrial oxidative stress and inflammation (Sawaddiruk et al. 2019).

### Gut microbiota disturbances

In FM, dysregulation of the gut-brain axis is thought to be involved in central sensitization, mood disturbances, and cognitive deficits *via* immune, endocrine, and neural pathways (Dipalma et al. 2025; Kishore et al. 2026). FM patients often experience gastrointestinal issues, such as irritable bowel syndrome, and their gut microbiota composition differs from that of healthy individuals (Sivri et al. 1996; Minerbi et al. 2019). This dysbiosis is characterized by a deficiency of beneficial bacteria, an increased abundance of harmful bacteria, as well as changes in bacterial metabolites, like short-chain fatty acids. These perturbations contribute to altered intestinal permeability, allowing harmful substances to enter the circulation and initiate an immune response, with subsequent inflammatory cascades (Clauw et al. 2024; Palma-Ordóñez et al. 2024; Kishore et al. 2026). Interestingly, a diet rich in acetylated high-amylose maize starch has been shown to shift the gut microbiome toward acetate-producing bacteria, thereby reducing spinal microglia activation, dorsal horn hyperexcitability, and pain hypersensitivity in a rat model of reserpine-induced FM (Chen et al. 2026). Additionally, gut dysbiosis may promote an imbalance between glutamate and GABA, along with serotonin deficiency (Palma‐Ordóñez, Moreno‐Fernández et al. 2024, Kishore et al. 2026). Transplantation of fecal microbiota from FM patients into germ-free mice induced pain hypersensitivity. Conversely, replacement of the FM microbiota with that of healthy individuals substantially ameliorated pain in mice. In an open-label trial involving women with FM, transplantation of healthy microbiota was associated with reduced pain and improved quality of life (Cai et al. 2025). Moreover, findings from an RCT demonstrated that probiotic supplementation in female FM patients significantly improved pain, sleep quality, depression, and anxiety whereas prebiotic supplementation markedly improved pain and sleep quality (Aslan Çİn et al. 2024).

### Autoimmunity

Being a part of the peripheral nervous system, DRG contains neurons with unique physiological properties that facilitate the initiation of pain signals in response to diverse afferent stimuli. Activated satellite glial cells (SGCs), closely enveloping the cell bodies of sensory neurons in the DRG, release pro-inflammatory and pro-nociceptive mediators, thereby enhancing pain signaling (Hanani and Spray 2020; Findeisen et al. 2025). Growing evidence supports the pathogenic role of anti-SGC IgG antibodies in FM, highlighting the importance of the DRG and indicating a potential autoimmune contribution. Anti-SGC IgG antibodies were elevated in the serum and plasma of FM patients, correlating with disease severity, though levels vary between individuals. This suggests these autoantibodies may contribute to FM in a subset of patients (Fanton et al. 2023; Krock et al. 2023; Findeisen et al. 2025). Importantly, passive transfer of IgG from FM patients to mice resulted in sensory hypersensitivity and increased nociceptor excitability, whereas IgG-depleted serum had no effect. IgG from patients was consistently detected in mouse DRG but not in the spinal cord. It was primarily localized to SGCs and fiber tracts entering the DRG, accompanied by evidence of increased SGC activity (Goebel et al. 2021). Additionally, it has been observed that IgG autoantibodies from sera of 37% of FM patients bound to rat DRG neurons, while no binding was detected in healthy controls, with greater binding to SGCs correlated with increased symptom severity (Seefried et al. 2025). However, targeting circulating IgG with rozanolixizumab, a monoclonal antibody against the neonatal Fc receptor (FcRn), did not demonstrate a clinically meaningful improvement in FM-related pain interference in an RCT (NCT05643794).

## Therapeutic strategies in fibromyalgia

Therapeutic strategies in FM encompass both non-pharmacological and pharmacological approaches.

### Non-pharmacological approaches

#### Patient education

Patient education is considered the initial step in the management of FM. It includes educational activities planned by healthcare professionals that provide patients with basic information about the disease, its associated symptoms, available therapeutic strategies, and stress management. These activities aim to improve treatment adherence and encourage patients’ active participation in the management plan (García Ríos et al. 2019; Musekamp et al. 2019).

#### Psychological therapies

Psychological therapies comprise cognitive behavioral therapy (CBT), education, relaxation/biofeedback, and mindfulness-based treatment. These interventions provide an adjunctive benefit in the management of FM by targeting the psychological mechanisms underlying pain perception, sleep disturbance, depression, and anxiety. Specifically, CBT, the most effective psychological therapy, addresses avoidance of painful experiences and unpleasant thought patterns, thereby reducing their adverse psychological and behavioral consequences (Jones et al. 2024).

#### Exercise therapy

Exercise therapy has a positive impact on the management of FM due to improvements in pain, sleep quality, anxiety, and overall health. These beneficial effects are attributed to aerobic exercise and other interventions including Qi Gong, Tai Chi, yoga, or pool therapy (Jones et al. 2024; Wang et al. 2026).

#### Sleep management

Sleep hygiene education includes caffeine intake, smoking, bed comfort, room temperature, lighting, noise, and daytime sleeping habits. It can improve sleep quality and alleviate pain associated with FM (Okul and Fertelli 2025).

#### Dietary modifications

Nutritional strategies may involve dietary components and supplements, such as vegetables, extra-virgin olive oil, acetyl-L-carnitine, coenzyme Q10, and a combination of vitamins C and E (Jones et al. 2024).

### Pharmacological approaches

Pharmacological approaches should be individualized to target each patient’s most prominent symptoms, including pain, sleep disturbance, anxiety, and depression, and can be used in conjunction with non-pharmacological interventions to optimize patient outcomes. The current Food and Drug Administration (FDA)-approved drugs for FM are pregabalin, duloxetine, and milnacipran. Pregabalin, an antiepileptic drug, binds to the α2δ subunit of voltage-gated calcium channels and reduces calcium influx at presynaptic nerve terminals, thereby decreasing the release of excitatory neurotransmitters such as glutamate and contributing to improvements in pain, anxiety, and sleep quality. Duloxetine and milnacipran are serotonin and norepinephrine reuptake inhibitors (SNRIs), used in patients with comorbid depression (Ablin and Häuser 2016; Jones et al. 2024).

On 15 August 2025, FDA approved Tonmya (cyclobenzaprine hydrochloride sublingual tablets) for FM, demonstrating efficacy in improving pain and sleep quality (Corp 2025; Hujjat et al. 2025). In clinical practice, several off-label medications are also commonly used. Amitriptyline, a tricyclic antidepressant, is commonly used off-label to treat FM patients with comorbid sleep disturbance. Likewise, gabapentin, another antiepileptic drug, exhibits effects similar to those of pregabalin (Jones et al. 2024). Although FM is a complex syndrome, only four drugs have been approved by the FDA, and all of which primarily provide symptomatic relief. Many patients experience inadequate improvement or significant side effects from these medications, often leading them to discontinue treatment. Accordingly, there is a critical need for further research to improve therapeutic options for FM (Ablin and Buskila 2010; González-Flores et al. 2023; Jones et al. 2024).

## Repurposed drugs in preclinical and clinical studies of fibromyalgia

### NMDA receptor antagonists

Glutamate is a major driver of central sensitization through activation of NMDA receptor (Atta et al. 2023a, b). A systematic review reported that ketamine, an NMDA receptor antagonist, exhibited short-term analgesic effects following intravenous infusion in patients with FM. Thus, further studies with longer follow-up periods are still needed to better define its efficacy (de Carvalho and de Sena 2024). Memantine, another NMDA receptor antagonist, significantly alleviated pain and improved quality of life in FM patients, with evidence emerging from both an RCT and an open-label study (Olivan-Blázquez et al. 2014; Khalid et al. 2015). Moreover, low-dose dextromethorphan reduced baseline pain in a pilot clinical trial of FM (Mueller et al. 2021). However, these findings warrant confirmation in larger, well-controlled clinical studies.

### Neurokinin-1 receptor antagonists

Notably, SP is implicated in pain transmission and central sensitization by activating NK-1R (Siracusa et al. 2021; Chen et al. 2025). The SP/NK-1R signaling also contributes to the activation of M1 microglia (Yang et al. 2022; Atta et al. 2023a, b). In addition, elevated levels of SP were detected in the cerebrospinal fluid (CSF) of FM patients, suggesting its involvement in the pathophysiology of the disorder (Russell et al. 1994). Casopitant, an NK-1R antagonist, was evaluated in a pilot RCT (NCT00264628) involving patients with FM and comorbid depression; however, evidence supporting its efficacy wasn’t established. Despite the mechanistic relevance of SP/NK-1R signaling in FM pathophysiology, this pathway remains underexplored, and key agents such as aprepitant have not yet been investigated.

### GABAergic drugs

Interestingly, GABA receptor-mediated inhibitory signaling is involved in suppressing central sensitization (Atta et al. 2023a, b). Benzodiazepines, positive allosteric modulators (PAMs) of GABA-A receptors, have insufficient evidence to support their efficacy in FM and are generally discouraged due to potential adverse effects (Corrigan et al. 2012; Macfarlane et al. 2017; Giorgi et al. 2024). Short-acting benzodiazepines may improve initial insomnia. Additionally, non-benzodiazepine sedative-hypnotics such as zopiclone and zolpidem can enhance sleep and reduce fatigue (Giorgi et al. 2024). In RCTs, the sodium salt of gamma-hydroxybutyrate (GHB), a GABA-B receptor agonist, significantly improved pain and sleep quality in patients with FM (Russell et al. 2011; Spaeth et al. 2012). However, the use of systemic GABA-B receptor agonists, including GHB and baclofen, is restricted due to their undesirable side effects. One possible approach to avoid GABA-B receptor agonist-induced side effects is the use of PAMs. Accordingly, ASP8062, a GABA-B receptor PAM, was shown to restore decreased muscle pressure threshold and ameliorate sleep disturbance and motor coordination deficits in a rat model of reserpine-induced FM (Murai et al. 2019). Conversely, it did not exhibit clinically meaningful analgesic effects among FM patients in an RCT (NCT03092726).

### Antiepileptic drugs

Antiepileptic drugs reduce excitatory neurotransmission and/or enhance inhibitory neurotransmission, which may help to attenuate central sensitization in FM. Pregabalin is approved by the FDA for FM, while gabapentin is commonly used in patients who cannot tolerate other medications (Giorgi et al. 2024; Jones et al. 2024). Mirogabalin ameliorated anxiety-like behaviors and cognitive impairment in a rat model of acidic saline-induced FM (Murasawa et al. 2020, 2021). By contrast, it failed to demonstrate a significant pain reduction among FM patients in an RCT (Arnold et al. 2019). A systematic review evaluating several antiepileptic drugs, such as clonazepam, phenytoin, valproate, carbamazepine, lamotrigine, oxcarbazepine, topiramate, and lacosamide reported little to no evidence supporting their efficacy in reducing pain (Wiffen et al. 2013).

### Antidepressant drugs

Most clinically used antidepressant drugs inhibit the reuptake of serotonin and/or norepinephrine, thereby facilitating descending inhibitory pain pathways. The SNRIs, such as duloxetine and milnacipran, are approved by the FDA for treatment of FM. In contrast, the selective serotonin reuptake inhibitors (SSRIs) are generally less effective than SNRIs in managing chronic pain, but they remain effective in treating comorbid depression and anxiety and may provide modest improvement in overall quality of life (Ablin and Häuser 2016; Jones et al. 2024). Vortioxetine, an atypical antidepressant, ameliorated tactile allodynia and anxiety-like behaviors in a mouse model of reserpine-induced FM (Sałat and Furgała-Wojas 2021). Additionally, a prospective study demonstrated the potential benefits of vortioxetine in cognitive symptoms and distress tolerance, although duloxetine was more effective in reducing depression, anxiety, and somatic amplification in patients with FM (Aker and Öke 2025). A retrospective cohort study documented that low-dose mirtazapine, an atypical antidepressant, was less effective than duloxetine in the management of FM (Mehta et al. 2022). Further, an open-label study reported that trazodone, an atypical antidepressant, significantly improved sleep quality, anxiety, depression, and pain interference with daily activities in FM patients (Morillas-Arques, Ma Rodriguez-Lopez et al. 2010). Moreover, the combination of trazodone and pregabalin was associated with further improvements in FM severity, depression, and pain interference with daily activities (Calandre et al. 2011).

### Opioids

Pure µ-opioid receptor agonists, including codeine, fentanyl, and oxycodone, are typically not recommended due to their limited clinical efficacy and the potential risk of opioid-induced hyperalgesia (Jones et al. 2024). By contrast, tramadol, a weak µ-opioid receptor agonist that also inhibits serotonin and norepinephrine reuptake, exhibited antiallodynic effect in a rat model of reserpine-induced FM (Kaneko et al. 2014). The combination of tramadol and paracetamol demonstrated prominent analgesic effects and improved quality of life among FM patients in an RCT (Bennett et al. 2003). In addition, a pilot trial involving FM patients reported that low-dose naltrexone (LDN) alleviated pain and overall symptoms, alongside reducing plasma levels of pro-inflammatory cytokines (Parkitny and Younger 2017). Further, systematic reviews and a meta-analysis of RCTs indicated that LDN exerted analgesic effects in patients with FM; however, a subsequent re-analysis suggested lower efficacy than previously mentioned (Yang et al. 2023; Vatvani et al. 2024; Bruun et al. 2026). Notably, a recent well-powered RCT failed to confirm the promising efficacy of LDN observed in previous studies, as it showed no clinically meaningful improvement in pain intensity (Rodríguez-Freire et al. 2026).

### Cannabinoids

The endocannabinoid system is known to be involved in pain modulation, besides it has been hypothesized that FM may represent a state of endocannabinoid deficiency (Ablin and Häuser 2016). Cannabinoids mainly include two principal active components, tetrahydrocannabinol (THC) and cannabidiol (CBD). THC exerts analgesic effects through its psychoactive properties, while CBD exhibits anti-inflammatory and analgesic properties (Jones et al. 2024). Broad-spectrum cannabis oil with low THC concentration has been reported to ameliorate hyperalgesia, allodynia, and depression-like behaviors in a mouse model of reserpine-induced FM (Ferrarini et al. 2022). Similarly, THC-rich cannabis oil markedly alleviated pain and fatigue, alongside improving quality of life among FM patients in an RCT (Chaves et al. 2020). Additionally, the synthetic cannabinoid nabilone significantly reduced pain and improved quality of life in FM patients (Skrabek et al. 2008). Conversely, another RCT on nabilone demonstrated improvement in sleep quality without significant effects on pain, mood, or quality of life (Ware et al. 2010). Hence, further well-controlled studies are required to clarify the therapeutic role of nabilone in the management of FM.

### Dopamine receptor agonists

Dopaminergic system dysfunction has been linked to altered pain processing in FM (Albrecht et al. 2016; Ledermann et al. 2016). Ropinirole ameliorated tactile allodynia and depression-like behaviors in a mouse model of reserpine-induced FM (Sałat and Furgała-Wojas 2021). Notably, an RCT (NCT00256893) evaluating its efficacy did not demonstrate clear therapeutic benefits, whereas a separate case series showed significant improvements in pain, anxiety, and quality of life in patients with refractory FM (Garcia-Leiva et al. 2009). Likewise, pramipexole reduced hyperalgesia and allodynia as well as it ameliorated depression and anxiety-like behaviors in a mouse model of reserpine-induced FM, possibly through attenuation of oxidative stress (Martins et al. 2022). Clinically, pramipexole also demonstrated significant improvements in pain and functional status among FM patients in an open-label study and an RCT (Holman et al. 2004; Holman and Myers 2005). However, another RCT (NCT00689052) was terminated, and no detailed results were published. Despite consistent preclinical findings supporting dopaminergic modulation in FM models, clinical studies showed variable outcomes, emphasizing the need for larger, well-controlled trials. Moreover, ergot-derived dopamine agonists, such as bromocriptine and cabergoline, remain largely unexplored in FM.

### Melatonin receptor agonists

Melatonin, an endogenous hormone secreted by the pineal gland, promotes sleep in accordance with circadian rhythms (Ablin and Häuser 2016). Research suggested beneficial effects of melatonin in neuropathic pain and migraine, highlighting its potential role in the management of chronic pain conditions (Lin et al. 2017; Zeng et al. 2023; Mehramiri et al. 2024; Kilinc et al. 2025). In a rat model of reserpine-induced FM, melatonin improved skeletal muscle performance and downregulated NLRP3 (Favero et al. 2017). Additionally, it ameliorated reserpine-induced hyperalgesia, allodynia, and depression-like behaviors in rats, presumably by attenuating neuroinflammation, oxidative stress, and mitochondrial dysfunction (Fusco et al. 2019; Osama et al. 2026). Clinical evidence from an RCT demonstrated that melatonin alone or in combination with amitriptyline significantly reduced pain in patients with FM, while the combination therapy showed better improvement in functional status than either monotherapy (de Zanette et al. 2014). Further, preliminary clinical studies evaluating melatonin reported improvements in sleep quality, pain, anxiety, and quality of life, along with enhanced antioxidant capacity in FM patients (Castaño et al. 2018, 2019). Furthermore, the melatonergic agonist agomelatine significantly alleviated pain, depression, and anxiety in preliminary clinical studies of FM (Bruno et al. 2013; Calandre et al. 2014). Therefore, these outcomes warrant confirmation in larger, well-controlled clinical trials.

### Antidiabetic drugs

#### Glucagon-like peptide-1 receptor agonists

Glucagon-like peptide-1 (GLP-1) receptor agonists, originally developed for type 2 diabetes mellitus (T2DM), have recently gained attention for their beneficial effects in diabetic neuropathic pain and migraine (Jing et al. 2023; Zhang et al. 2023; Lee et al. 2024; Sićović et al. 2024; Braca et al. 2025). In a rat model of reserpine-induced FM, liraglutide improved skeletal muscle performance and enhanced pain threshold, possibly through its antioxidant effects (Hassan et al. 2025). Likewise, semaglutide ameliorated hyperalgesia and depression-like behaviors, in addition, it improved motor coordination in a rat model of reserpine-induced FM, presumably by attenuating neuroinflammation and promoting microglial polarization toward the M2 phenotype (Shafiek et al. 2025). Nevertheless, these findings warrant validation in well-controlled clinical studies.

#### Insulin sensitizers

Pioglitazone, a peroxisome proliferator-activated receptor gamma (PPAR-γ) agonist commonly used as an insulin sensitizer in T2DM, was shown to exhibit analgesic, anti-inflammatory, and antioxidant effects across multiple preclinical studies of neuropathic pain (Griggs et al. 2015; Jia et al. 2016; Khasabova et al. 2019). In the context of FM, it improved skeletal muscle functions and promoted antioxidant defense mechanisms in reserpine-treated rats (Hassan et al. 2021). Similarly, metformin, an insulin sensitizer commonly used in T2DM, ameliorated hyperalgesia, allodynia, and depression-like behaviors in a mouse model of reserpine-induced FM, possibly through attenuation of neuroinflammation and modulation of neurotransmitter levels, including increased serotonin and norepinephrine and reduced glutamate levels (Abotaleb et al. 2024). Further, it reduced oxidative stress and mitochondrial dysfunction in fibroblasts from FM patients (Alcocer-Gómez et al. 2015). Moreover, metformin significantly improved pain, fatigue, sleep quality, and depression among FM patients in a small pilot trial, accompanied by enhanced AMPK activation, reduced NLRP3 expression levels in BMCs, and decreased serum levels of IL-1β and IL-18 (Bullón et al. 2016). However, further well-controlled trials are required to confirm these findings.

### Nonsteroidal anti-inflammatory drugs

Nonsteroidal anti-inflammatory drugs are generally not effective in the management of FM, as the condition is primarily driven by central sensitization rather than muscle or joint inflammation. However, they may be used in cases of mixed-type pain in combination with conventional FM medications (Sarzi-Puttini et al. 2010; Giorgi et al. 2024). In this regard, the combination of famciclovir and celecoxib significantly improved pain, fatigue, sleep quality, depression, and anxiety among FM patients in an RCT, supporting the hypothesis that herpesvirus infections may contribute to the pathogenesis of FM (Pridgen et al. 2017, 2021).

Finally, we summarized the preclinical and clinical studies investigating repurposed drugs in FM (Tables 1 and 2).

**Table 1:** Repurposed Drugs in Preclinical Studies of Fibromyalgia

Repurposed drug	Experimental model	Key findings	Proposed mechanisms	References
ASP8062 (GABA-B receptor positive allosteric modulator)	Reserpine rat model of FM	Reversal of reduced muscle pressure threshold Improvements in motor coordination and sleep quality	Positive allosteric modulation of GABA-B receptor	(Murai et al. 2019)
Mirogabalin (α2δ-1 calcium channel ligand)	Acidic saline rat model of FM	Amelioration of anxiety-like behaviors and cognitive impairment		(Murasawa et al. 2020, 2021)
Vortioxetine (Serotonin receptor modulator and reuptake inhibitor)	Reserpine mouse model of FM	Amelioration of tactile allodynia and anxiety-like behaviors		(Sałat and Furgała-Wojas 2021)
Tramadol (Weak µ-opioid receptor agonist and SNRI)	Reserpine rat model of FM	Amelioration of allodynia	Partial opioid receptor agonism	(Kaneko et al. 2014)
Ropinirole (Dopamine receptor agonist)	Reserpine mouse model of FM	Amelioration of tactile allodynia and depression-like behaviors		(Sałat and Furgała-Wojas 2021)
Pramipexole (Dopamine receptor agonist)	Reserpine mouse model of FM	Reduction in hyperalgesia and allodynia Amelioration of depression- and anxiety-like behaviors	Antioxidant effects	(Martins et al. 2022)
Melatonin (Melatonin receptor agonist)	Reserpine rat model of FM	Amelioration of hyperalgesia, allodynia, and depression-like behaviors Improvement in skeletal muscle performance	Anti-inflammatory effects NLRP3 downregulation Antioxidant effects Attenuation of mitochondrial dysfunction	(Favero et al. 2017; Fusco et al. 2019; Osama et al. 2026)
Galantamine (Acetylcholinesterase inhibitor and α7-nAChR positive allosteric modulator)	Reserpine rat model of FM	Amelioration of hyperalgesia, allodynia, and depression-like behaviors Improvement in motor coordination	Positive allosteric modulation of α7-nAChR Enhancement of descending inhibitory pain pathways Anti-inflammatory effects Promotion of microglial polarization toward the M2 phenotype Antioxidant effects	(Atta et al. 2023a, b)
Liraglutide (GLP-1 receptor agonist)	Reserpine rat model of FM	Enhancement of pain threshold Improvement in skeletal muscle performance	Antioxidant effects	(Hassan et al. 2025)
Semaglutide (GLP-1 receptor agonist)	Reserpine rat model of FM	Amelioration of hyperalgesia and depression-like behaviors Improvement in motor coordination	Anti-inflammatory effects Promotion of microglial polarization toward the M2 phenotype	(Shafiek et al. 2025)
Pioglitazone (PPAR-γ agonist/ insulin sensitizer)	Reserpine rat model of FM	Improvement in skeletal muscle functions	PPAR-γ receptor agonism Antioxidant effects	(Hassan et al. 2021)
Metformin (AMPK activator/ insulin sensitizer)	Reserpine mouse model of FM	Amelioration of hyperalgesia, allodynia, and depression-like behaviors	Enhancement of descending inhibitory pain pathways Anti-inflammatory effects	(Abotaleb et al. 2024)

**Table 2:** Repurposed drugs in clinical studies of fibromyalgia

Repurposed drug	Key findings	Evidence strength	Safety and Tolerability	Limitations	References
Memantine (NMDA receptor antagonist)	Significant improvements in pain and quality of life	RCT and open-label study (*n* = 63, 6 months; *n* = 30, 3 months)	Well tolerated	Preliminary evidence requiring confirmation in larger well-controlled trials	(Olivan-Blázquez et al. 2014; Khalid et al. 2015)
GHB (GABA-B receptor agonist)	Significant improvements in pain and sleep quality	2 RCTs (*n* = 548, 14 weeks; *n* = 573, 14 weeks)	Encouraging safety profile	Short duration Lack of long-term efficacy and safety data	(Russell et al. 2011; Spaeth et al. 2012)
Mirogabalin (α2δ-1 calcium channel ligand)	No significant pain reduction	3 RCTs (*n* = 1293, 13 weeks; *n* = 1270, 13 weeks; *n* = 1301 ,13 weeks)	Well tolerated	Limited clinical efficacy	(Arnold et al. 2019)
Vortioxetine (Serotonin receptor modulator and reuptake inhibitor)	Vortioxetine improved cognitive symptoms and distress tolerance, whereas duloxetine showed greater effects on depression, anxiety, and somatic amplification	Prospective comparative study (*n* = 100, 6 months)	Well tolerated	Lack of randomization	(Aker and Öke 2025)
Trazodone (Serotonin receptor antagonist and reuptake inhibitor)	Significant improvements in sleep quality, anxiety, depression, and pain interference with daily activities	Open-label study (*n* = 66, 12 weeks)	The most frequent and severe adverse event was tachycardia (21.2%)	Open-label design without control group Preliminary evidence requiring confirmation in larger and longer well-controlled trials	(Morillas-Arques, Ma Rodriguez-Lopez et al. 2010)
Low-dose naltrexone (Opioid receptor antagonist)	No clinically meaningful improvement in pain intensity	RCT (*n* = 98, 12 months)	Well tolerated	Limited clinical efficacy	(Rodríguez-Freire et al. 2026)
Nabilone (Cannabinoid receptor agonist)	One RCT showed significant improvements in pain and quality of life, whereas another found no significant benefits Enhanced sleep quality	2 RCTs (*n* = 40, 4 weeks; *n* = 29, 2 weeks)	Encouraging safety profile	Conflicting findings requiring confirmation in larger and longer well-controlled trials	(Skrabek et al. 2008; Ware et al. 2010)
Ropinirole (Dopamine receptor agonist)	Significant improvements in pain, anxiety, and quality of life	Case series (*n* = 23, 12 weeks)	High rate of adverse reactions (74%) Drug-related withdrawal (22%)	Small uncontrolled case series Short duration Safety concerns	(Garcia-Leiva et al. 2009)
Pramipexole (Dopamine receptor agonist)	Significant improvements in pain and functional status	RCT and open-label study (*n* = 60, 14 weeks; *n* = 22, 2 months)	Encouraging safety profile	Preliminary evidence requiring confirmation in larger and longer well-controlled trials	(Holman et al. 2004; Holman and Myers 2005)
Melatonin (Melatonin receptor agonist)	Significant reduction in pain Improvements in sleep quality, anxiety, and quality of life Enhancement of total antioxidant capacity	RCT and preliminary studies (*n* = 63, 6 weeks; *n* = 33, 10 days; *n* = 36, 10 days)	Well tolerated	Preliminary evidence requiring confirmation in larger and longer well-controlled trials	(de Zanette et al. 2014; Castaño et al. 2018, 2019)
Agomelatine (Melatonin receptor agonist and 5-HT2 receptor antagonist)	Significant improvements in pain, depression, and anxiety	Open-label and uncontrolled pilot studies (*n* = 15, 12 weeks; *n* = 23, 12 weeks)	Well tolerated	Preliminary evidence requiring confirmation in larger and longer well-controlled trials	(Bruno et al. 2013; Calandre et al. 2014)
Metformin (AMPK activator/ insulin sensitizer)	Significant improvements in pain, fatigue, sleep quality, and depression AMPK activation and NLRP3 downregulation	Small pilot trial (*n* = 6, 7 months)	Well tolerated	Very small sample size Preliminary evidence requiring confirmation in larger well-controlled trials	(Bullón et al. 2016)
Combination of famciclovir and celecoxib (Antiviral + NSAID)	Significant improvements in pain, fatigue, sleep quality, depression, and anxiety	RCT with a post hoc analysis (*n* = 143, 16-weeks)	Encouraging safety profile	Short duration Fixed-dose combination prevents assessment of the individual	(Pridgen et al. 2017, 2021)

## Conclusions

Future research frontiers in FM should consider comorbidities and drug repurposing from mechanistically related pain conditions such as migraine and neuropathic pain. In parallel, efforts should target central sensitization through multiple approaches, including inhibition of excitatory neurotransmission, enhancement of inhibitory neurotransmission, and elevation of serotonin as well as norepinephrine levels to strengthen descending inhibitory pain pathways.

Additionally, targeting gut dysbiosis and suppression of neuroinflammation by inhibiting NLRP3 inflammasome and promoting microglial M2 and astrocytic A2 polarization, along with reducing oxidative stress and enhancing mitochondrial biogenesis may represent promising approaches for drug discovery in FM. However, some of these approaches are largely based on preclinical evidence rather than robust clinical validation in patients with FM. Thus, further investigation in well-designed clinical studies is required, as most current clinical research primarily focuses on safety and efficacy, with limited emphasis on elucidating the underlying mechanisms of action.

A translational gap exists between preclinical and clinical findings, since several drugs showing promising effects in preclinical studies failed to demonstrate consistent efficacy in clinical trials. This discrepancy may be partly attributed to the inability of experimental models to fully capture the heterogeneity of FM. Further, conflicting outcomes were observed across different clinical studies evaluating the same drug. Moreover, repurposed drugs from different classes showed positive outcomes in preliminary clinical studies and RCTs; however, long-term efficacy remains insufficiently studied, highlighting the need for larger, well-designed trials with extended follow-up to better establish their efficacy. Therefore, addressing these challenges is necessary in future research to develop effective therapies for FM.

## Data Availability

No datasets were generated or analysed during the current study.

## Abbreviations

ACR: American college of rheumatology
AMPA: α-amino-3-hydroxy-5-methyl-4-isoxazolepropionic acid
AMPK: Adenosine monophosphate-activated protein kinase
ASC: Apoptosis-associated speck-like protein containing CARD
ATP: Adenosine triphosphate
BDNF: Brain-derived neurotrophic factor
BMCs: Blood mononuclear cells
CBD: Cannabidiol
CBT: Cognitive behavioral therapy
CNS: Central nervous system
CoQ10: Coenzyme Q10
CSF: Cerebrospinal fluid
DRG: Dorsal root ganglia
ERK: Extracellular signal-regulated kinase
FcRn: Fc receptor
FDA: Food and Drug Administration
FM: Fibromyalgia
GABA: Gamma-aminobutyric acid
GHB: Gamma-hydroxybutyrate
GLP-1: Glucagon-like peptide-1
IL-1β: Interleukin-1β
iNOS: Inducible nitric oxide synthase
JNK: c-Jun-N-terminal kinase
LDN: Low-dose naltrexone
MAPKs: Mitogen-activated protein kinases
NF-κB: Nuclear factor-kappa B
NK-1R: Neurokinin-1 receptor
NLRP3: NOD-like receptor protein 3
NMDA: N-methyl-D-aspartate
NOX2: NADPH oxidase 2
NRF 1: Nuclear respiratory factor 1
NSAID: Non-steroidal anti-inflammatory drug
PAMs: Positive allosteric modulators
PGC-1α: Peroxisome proliferator-activated receptor-gamma coactivator 1-alpha
PPAR: Peroxisome proliferator-activated receptor
RCT: Randomized controlled trial
ROS: Reactive oxygen species
SGCs: Satellite glial cells
SNRIs: Serotonin and norepinephrine reuptake inhibitors
SP: Substance P
SSRIs: Selective serotonin reuptake inhibitors
SSS: Symptom severity scale
T2DM: Type 2 diabetes mellitus
TFAM: Mitochondrial transcription factor A
THC: Tetrahydrocannabinol
TLR4: Toll-like receptor-4
TNF-α: Tumor necrosis factor-alpha
WPI: Widespread pain index
α7-nAChR: Alpha-7 nicotinic acetylcholine receptor
